# Persistent increase in cardiac troponin T at hospital discharge predicts repeat hospitalization in patients with acute decompensated heart failure

**DOI:** 10.1371/journal.pone.0173336

**Published:** 2017-04-05

**Authors:** Seiji Takashio, Toshiyuki Nagai, Yasuo Sugano, Satoshi Honda, Atsushi Okada, Yasuhide Asaumi, Takeshi Aiba, Teruo Noguchi, Kengo F. Kusano, Hisao Ogawa, Satoshi Yasuda, Toshihisa Anzai, on behalf of the NaDEF Investigators

**Affiliations:** Department of Cardiovascular Medicine, National Cerebral and Cardiovascular Center, Osaka, Japan; Osaka University Graduate School of Medicine, JAPAN

## Abstract

**Background:**

High-sensitive cardiac troponin T (hsTnT) is a sensitive biomarker of myocardial damage and predictor of acute decompensated heart failure (ADHF). However, there is little information on changes over time in hsTnT level during ADHF management. The aim of this prospective study was to evaluate changes in hsTnT levels between admission and at discharge in patients with ADHF, and identify factors that determine such levels and their prognostic significance.

**Methods and results:**

We evaluated 404 ADHF patients with abnormal hsTnT levels (≥0.0135 ng/ml) on admission. The median (interquartile ranges) hsTnT levels on admission, at discharge, and percent changes in hsTnT levels were 0.038 (0.026 to 0.065), 0.032 (0.021 to 0.049) ng/ml, and -12.0 (-39.8 to 7.4) % respectively. The numbers of patients with falling (hsTnT decrease > -15%), stable (hsTnT change between -15 and +15%) and rising (hsTnT increase > +15%) hsTnT level at discharge were 190, 146, and 68, respectively. The percent change in B-type natriuretic peptide (BNP) levels was greater in the falling group, compared to the stable (p<0.001) and rising groups (p<0.001). Changes in hsTnT levels correlated significantly with changes in BNP levels (ρ = 0.22, p<0.001). Multivariate Cox regression analysis identified rising or stable hsTnT at discharge as a significant predictor of heart failure-related rehospitalization (hazard ratio: 1.69; 95% confidence interval: 1.06 to 2.70; p = 0.03).

**Conclusions:**

Persistent increase in hsTnT levels at discharge correlated with inadequate decrease of BNP levels, and was a predictor of poor clinical outcome, with repeat heart failure hospitalizations.

## Introduction

Persistent and modest elevation in circulating cardiac troponin level is frequently observed in patients with heart failure (HF) and is considered to represent ongoing subclinical myocardial damage [1]. Furthermore, cardiac troponin I and T are used as biomarkers for myocardial damage and predictors of acute and chronic HF [2–5]. Various mechanisms have been proposed for the persistent hypertroponinemia in HF, including myocardial ischemia, increased wall stress, myocyte damage from inflammatory cytokines and/or oxidative stress, neurohormonal activation, and coronary microvascular dysfunction [1,6]. Since the exacerbation of HF causes additional myocardial damage due to activation of the above factors, cardiac troponin levels are significantly elevated in patients with acute decompensated heart failure (ADHF) compared to the compensated state [7]. Therefore, therapeutic strategies that can reduce of cardiac troponin level during ADHF management are desired.

Several studies have reported that a serial increase in cardiac troponin levels during ADHF management is a predictor of poor clinical outcome in such patients [8–10]. However, there is little information on cardiac troponin levels measured in both on admission and at discharge, and the factors associated with the change over time in cardiac troponin levels. The present study was designed to determine differences in high-sensitivity cardiac troponin T levels (hsTnT) measured on admission to and at discharge from the hospital, and identify the factors that determine such levels and their prognostic significance.

## Materials and methods

### Study design

Data from the NaDEF (National cerebral and cardiovascular center acute DEcompensated heart Failure) registry, which were obtained between January 2013 and March 2015, were retrospectively analyzed. The NaDEF registry is a single-center, observational, on-going, prospective cohort that includes all patients requiring hospitalization to our institution for the first time with a diagnosis of ADHF by at least two experienced cardiologists according to the Framingham ADHF criteria [11], and follow-up was performed at 3, 6, 12, and 24 months after discharge by direct contact with patients or their physicians in the hospital or outpatient clinic, telephone interview of patients or, if deceased, of family members, and mail, by dedicated coordinators and investigators. In this study, because patient information was anonymized and de-identified prior to analysis, written informed consent was not obtained from each patient. However, we publicized the study by posting a summary of the protocol (with an easily understood description) on the website of the National Cerebral and Cardiovascular Center; the notice clearly informed patients of their right to refuse enrollment. These procedures for informed consent and enrollment were in accordance with the detailed regulations regarding informed consent described in the guidelines, and this study, including the procedure for enrollment, has been approved by the Institutional Review Board of the National Cerebral and Cardiovascular Center (M22-025), and registered under the Japanese UMIN Clinical Trials Registration (UMIN000017024).

### Patient population

We evaluated 651 consecutive patients with ADHF enrolled in the NaDEF registry. Of these, 247 were excluded due to the following reasons: missing hsTnT values (n = 133), acute coronary syndrome (n = 34), revascularization procedure or surgical intervention during hospitalization (n = 22), end-stage renal dysfunction on hemodialysis (n = 9), myocarditis (n = 3), and abnormally high hsTnT level (4.15 ng/ml) due to suspected acute coronary syndrome at discharge (n = 1). Patients with hsTnT levels below the 99th percentile upper reference limit (0.0135 ng/ml) on admission (n = 45) who were confirmed to be free of overt myocardial injury, were also excluded. The latter patients were excluded since the study focused on improvement of myocardial damage during ADHF management (patients with normal hsTnT levels on admission do not have overt myocardial injury). Furthermore, while present changes in hsTnT levels fluctuated widely, the net changes in hsTnT levels were very small at low hsTnT levels. Consequently, we prospectively evaluated the data of 404 ADHF patients with abnormal hsTnT levels (≥0.0135 ng/ml) on admission.

### Biomarker assays and measurement of hsTnT change

Blood samples were collected from all study patients on admission and at discharge and analyzed for serum cardiac troponin T levels using the Elecsys 2010 Troponin T hs STAT kit (Roche Diagnostics, Switzerland). The lower limit of detection is 0.003 ng/ml with a reported 99th percentile value in apparently healthy individuals of 0.0135 ng/ml. At the 99th percentile value, the coefficient of variation was 9% by Elecsys 2010 analyzer [12]. Plasma B-type natriuretic peptide (BNP) levels were also measured using the MI02 Shionogi BNP kit (Shionogi, Osaka, Japan). Changes in hsTnT levels were described as differences between hsTnT levels on admission and at discharge. Based on these values and considering biologic variability [13], the study patients were divided into the rising group (hsTnT change increase > +15%), stable group (hsTnT change between -15 and +15%), falling group (hsTnT change decrease > -15%), and also into the normalized group (hsTnT <0.00135 ng/ml at discharge) and persistently abnormal group (hsTnT ≥ 0.00135 ng/ml, both on admission and at discharge).

### Prognostic endpoint

For our analysis, the patients were followed from admission. Death from any cause and HF-related rehospitalization were set as the study endpoints. We analyzed the prognostic significance of changes in hsTnT levels in the first event of all-cause death, cardiovascular-related death and HF-related rehospitalization.

### Statistical analysis

Normally distributed data were presented as mean±SD while data of variables with skewed distribution were expressed as median with interquartile ranges. Differences between groups were examined by the Student’s *t*-test or the Mann-Whitney U test for unpaired data. Continuous variables were compared by analysis of variance or by the nonparametric Kruskal-Wallis test for nonnormally distributed data in cases where there are more than two groups. Categorical values were presented as numbers (percentage) and compared by the chi-square test or Fisher exact test as appropriate. The linear relationships between changes in hsTnT levels and key variables were examined using Spearman’s rank correlation test. Univariate logistic regression analysis was performed to identify significant parameters for stable or rising hsTnT levels at discharge. Then, multivariate logistic regression analysis was performed using the forced inclusion model that included the following parameters: age, sex, atrial fibrillation, prior HF hospitalization, hemoglobin, hsTnT and BNP level on admission, and increase of creatinine. The Hosmer-Lemeshow statistic was applied to assess model calibration. Kaplan-Meier curve for all-cause death, cardiovascular death and HF-related rehospitalization were compared among the three categories of patients with either rising, stable, or falling group, and between normalized and persistently abnormal groups. Univariate and multivariate Cox regression analyses were also used to assess the prognostic association of changes in hsTnT. A two-tailed value of *P*<0.05 was considered statistically significant. All statistical analyses were performed with The SPSS, version 19 (SPSS Inc, Chicago, IL).

## Results

### Changes in hsTnT levels

**Fig 1:**
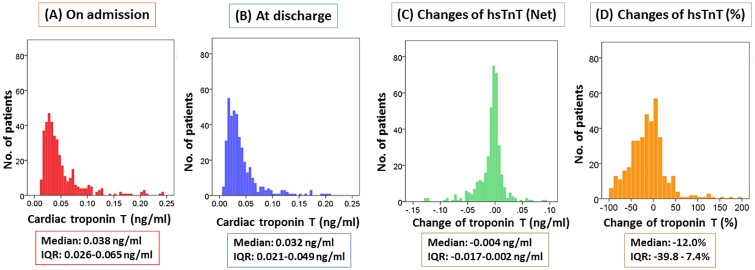
Distribution of hsTnT level on admission to the hospital (A), at discharge (B), changes in hsTnT levels (net) (C) and changes in hsTnT levels (percent) (D). The median (interquartile ranges) high sensitive cardiac troponin T (hsTnT) levels on admission, at discharge, and changes in hsTnT levels (net) were 0.038 (0.026 to 0.065), 0.032 (0.021 to 0.049), and -0.004 (-0.017 to 0.002) ng/ml, respectively. The percent change of hsTnT was -12.0 (-39.8 to 7.4) %.

Fig 1 shows the distribution of hsTnT levels in the 404 ADHF patients. Dataset of this study is available from the S1 file. The median hsTnT levels on admission and discharge were 0.038 (0.026 to 0.065) and 0.032 (0.021 to 0.049) ng/ml, respectively. The median values of the net and percent change in hsTnT were -0.004 (-0.017 to 0.002) ng/ml and -12.0 (-39.8 to 7.4) %, respectively. The median percent change in BNP was -59.1 (-76.3 to -33.7) %.

The numbers (percentages) of falling (hsTnT decrease > -15%), stable (hsTnT change between -15 and +15%) and rising groups (hsTnT increase > +15%) were 190 (47%), 146 (36%), and 68 (17%), respectively. Table 1 shows the clinical characteristics of these patients. Proportion of patients with history of atrial fibrillation, prior hospitalization for HF, changes in BNP and creatinine level were significantly higher, and diastolic blood pressure, heart rate, hsTnT and hemoglobin levels on admission were significantly lower among the stable or rising group compared to the falling group. The percent change in BNP was greater in the falling group (-67.0 [-78.8 to -44.5] %), compared to the stable (-52.9 [-72.7 to -30.4] %; p<0.001) and rising groups (-44.0 [-71.2 to -19.2] %; p<0.001). The use of inotropic agents and carperitide did not affect the change in hsTnT. The numbers of patients of the normalized group (hsTnT <0.00135 ng/ml at discharge) and those of the persistently abnormal group (hsTnT ≥ 0.00135 ng/ml at discharge) were 31 (8%) and 373 (92%), respectively.

**Table 1:** Clinical characteristics of patients of the hsTnT falling, stable or rising groups.

Variable	Overall (n = 404)	Falling group (n = 190)	Stable group (n = 146)	Rising group (n = 68)	P value
Age (years)	76.1±11.8	74.3±12.4	76.9±11.7	79.1±9.8*	0.009
Males	253 (63%)	121 (64%)	91 (62%)	41 (60%)	0.88
Body mass index (kg/m^2^)	23.2±4.2	23.5±4.5	22.8±3.8	23.2±4.1	0.29
Hypertension	282 (70%)	129 (68%)	99 (68%)	54 (79%)	0.17
Diabetes mellitus	146 (36%)	70 (37%)	50 (34%)	26 (38%)	0.82
Dyslipidemia	210 (52%)	92 (48%)	83 (57%)	35 (52%)	0.31
Chronic kidney disease	240 (59%)	106 (56%)	88 (60%)	46 (68%)	0.24
Atrial fibrillation	204 (51%)	79 (42%)	84 (58%) *	41 (60%) *	0.003
Prior HF hospitalization	202 (50%)	77 (41%)	85 (58%) *	40 (59%) *	0.002
Ischemic etiology	85 (21%)	44 (23%)	24 (16%)	17 (25%)	0.22
Use of inotropic agents	61 (15%)	31 (16%)	21 (14%)	9 (13%)	0.79
Use of carpertide	171 (42%)	88 (46%)	57 (39%)	26 (38%)	0.31
LVEF (%)	37.7±16.9	35.8±16.9	39.8±16.5	38.5±17.5	0.12
Duration of hospitalization (days)	20 (14–28)	20 (15–27)	19 (13–28)	20 (13–30)	0.41
Clinical signs on admission					
Systolic blood pressure (mmHg)	137±32	140±33	135±31	135±28	0.35
Diastolic blood pressure (mmHg)	79±21	82±22	76±21 *	76±20 *	0.03
Heart rate (bpm)	90±28	94±30	86±27 *	86±25 *	0.01
Medications on admission					
Loop diuretics	245 (61%)	100 (53%)	100 (69%) *	45 (66%)	0.008
β-blockers	218 (54%)	93 (49%)	85 (58%)	40 (59%)	0.16
ACE-I or ARB	215 (53%)	90 (47%)	83 (57%)	42 (62%)	0.07
Aldosterone antagonists	103 (26%)	40 (21%)	47 (32%)	16 (24%)	0.06
Laboratory data on admission					
Plasma BNP (pg/ml)	600 [335–1154]	677 [365–1289]	549 [320–899] *	522 [328–865]	0.047
Serum hsTnT (ng/ml)	0.038 [0.026–0.065]	0.048 [0.031–0.088]	0.034 [0.022–0.049] *	0.029 [0.022–0.046] *	<0.001
Hemoglobin (g/dl)	11.8±2.2	12.2±2.3	11.6±2.0 *	11.4±1.9 *	0.01
Serum sodium (mEq/L)	140±4	140±5	140±4	139±5	0.56
Estimated GFR (ml/min/1.73 m^2^)	44.5±21.2	43.5±19.4	45.9±20.7	44.5±26.5	0.57
Serum creatinine (mg/ml)	1.43±0.95	1.51±1.15	1.31±0.64	1.43±0.87	0.16
Total-bilirubin (mg/ml)	0.92±0.66	0.92±0.71	0.95±0.65	0.83±0.53	0.44
hs-CRP (mg/dl)	0.45 [0.14–1.25]	0.45 [0.13–1.70]	0.49 [0.17–1.13]	0.35 [0.14–0.97]	0.81
Change of laboratory data					
Changes in hsTnT (ng/ml)	-0.004 [-0.017–0.002]	-0.018 [-0.000- -0.013]	0.000 [-0.002–0.002] *	0.013 [0.007–0.028] *	<0.001
Changes in hsTnT (%)	-12.0 [-39.8–7.4]	-41.1 [-58.8- -24.8]	0.0 [-6.7–6.5] *	38.2 [22.9–56.0] *	<0.001
Changes in BNP (pg/ml)	-301 [–674– –123]	-410 [–951– –157]	-247 [–579– –96] *	-237 [–464– –58] *	<0.001
Changes in BNP (%)	-59.1 [-76.3- -33.7]	-67.0 [-78.8- -44.5]	-52.9 [-72.7- -30.4] *	-44.0 [-71.2- -19.2] *	<0.001
Changes in creatinine (mg/ml)	0.04 [-0.11–0.19]	0.01 [-0.02–0.15]	0.06 [-0.08–0.20] *	0.09 [-0.02–0.29] *	0.002
Changes in creatinine (%)	4.2 [-9.5–17.9]	1.0 [-12.4–14.7]	6.4 [-7.2–20.5] *	8.5 [-1.4–24.6] *	0.001
Increase in BNP¶	35 (9%)	11 (6%)	13 (9%)	11 (16%)*	0.04
Increase in creatinine§	247 (61%)	99 (52%)	100 (69%) *	48 (71%) *	0.002

Data are mean±SD, number of patients (%), or median [interquartile range]

* P<0.05 vs. falling group

¶ Changes in BNP level of ≥0 pg/ml

§ Changes in creatinine level of ≥0 mg/ml

ACE-I = angiotensin-converting enzyme inhibitor, ARB = angiotensin II receptor blocker, BNP = B-type natriuretic peptide, hsTnT = high sensitivity cardiac troponin T, HF = heart failure, hs-CRP = high-sensitivity C-reactive protein, GFR = glomerular filtration rate, LVEF = left ventricular ejection fraction

### Evaluation of factors associated with changes in hsTnT levels

Both the net and percent changes in hsTnT levels correlated positively with age, changes in BNP levels (both net and percent), and changes in creatinine (both net and percent), and negatively with serum hsTnT, plasma BNP and hemoglobin levels on admission (Table 2).

**Table 2:** Results of univariate linear regression analysis for changes in hsTnT levels.

Variable	Net change in hsTnT		Percent change hsTnT	
	ρ	p value	ρ	p value
Age (years)	0.120	0.02	0.155	0.002
Systolic blood pressure (mmHg)	-0.033	0.50	-0.048	0.40
LVEF (%)	0.085	0.11	0.097	0.07
Serum hsTnT (ng/ml)	-0.426	<0.01	-0.345	<0.01
Plasma BNP (pg/ml)	-0.153	0.002	-0.104	0.04
Hemoglobin (g/dl)	-0.114	0.02	-0.153	0.002
Serum sodium (mEq/L)	-0.012	0.81	-0.038	0.44
Estimated GFR (ml/min/1.73 m^2^)	0.074	0.14	0.002	0.97
Serum creatinine (mg/ml)	-0.089	0.07	-0.019	0.70
Total-bilirubin (mg/ml)	0.002	0.97	-0.024	0.62
hs-CRP (mg/ml)	-0.053	0.29	-0.030	0.55
Duration of hospitalization (days)	-0.047	0.34	-0.053	0.29
Changes in BNP level (pg/ml)	0.221	<0.001	0.195	<0.001
Changes in BNP level (%)	0.234	<0.001	0.249	<0.001
Changes in creatinine (mg/ml)	0.147	0.003	0.154	0.002
Changes in creatinine (%)	0.166	0.001	0.168	0.001

For abbreviations see Table 1.

### Factor associated with stable or rising hsTnT levels at discharge

We used univariate and multivariate logistic regression analyses to identify the parameters associated with stable or rising hsTnT levels at discharge. Multivariate analysis identified prior hospitalization for HF [odds ratio (OR): 2.10; 95% confidence interval (CI): 1.33 to 3.30, p = 0.001], serum hsTnT level at admission (per 0.001 ng/ml, OR: 0.99; 95% CI: 0.98 to 0.99, p<0.001) and increase in creatinine (OR: 2.08; 95% CI: 1.33 to 3.26, p = 0.001) as independent determinants of stable or rising hsTnT levels at discharge (Table 3). This model was reliable (p = 0.72, by the Hosmer-Lemeshow test).

**Table 3:** Results of univariate and multivariate logistic regression analyses for stable or rising hsTnT levels at discharge.

Variables	Univariate Analysis		Multivariate Analysis	
	p value	OR (95% CI)	p value	OR (95% CI)
Age (per 1 year)	0.006	1.02 (1.01–1.04)	0.26	1.01 (0.99–1.03)
Male	0.68	0.92 (0.61–1.38)	0.95	1.02 (0.64–1.61)
Hypertension (yes)	0.43	1.19 (0.78–1.82)		
Diabetes mellitus (yes)	0.78	0.94 (0.63–1.42)		
Dyslipidemia (yes)	0.18	1.31 (0.89–1.94)		
Chronic kidney disease (yes)	0.18	1.31 (0.88–1.96)		
Atrial fibrillation (yes)	0.001	1.97 (1.33–2.93)	0.15	1.39 (0.88–2.20)
Prior HF hospitalization (yes)	<0.001	2.06 (1.39–3.07)	0.001	2.10 (1.33–3.30)
Ischemic etiology (yes)	0.33	0.79 (0.49–1.27)		
Use of inotropic agents (yes)	0.52	0.84 (0.49–1.44)		
Use of carpertide (yes)	0.13	0.73 (0.49–1.09)		
Systolic blood pressure (mmHg)	0.15	1.00 (0.99–1.00)		
Duration of hospitalization (days)	0.78	1.00 (0.99–1.01)		
Hemoglobin (g/dl)	0.004	0.87 (0.79–0.96)	0.05	0.89 (0.80–1.00)
Serum hsTnT (per 0.001 ng/ml)	<0.001	0.99 (0.99–1.00)	<0.001	0.99 (0.98–0.99)
Plasma BNP (pg/ml)	0.003	1.00 (1.00–1.00)	0.31	1,00 (1.00–1.00)
Serum sodium (mEq/L)	0.84	1.00 (0.95–1.04)		
Estimated GFR (ml/min/1.73 m^2^)	0.34	1.01 (1.00–1.01)		
Serum creatinine (mg/ml)	0.10	0.83 (0.67–1.03)		
Total-bilirubin (mg/ml)	0.96	0.99 (0.74–1.33)		
hs-CRP (mg/ml)	0.25	0.96 (0.90–1.03)		
Increase in BNP (yes)	0.06	2.07 (0.98–4.34)		
Increase in creatinine (yes)	0.001	2.06 (1.37–3.10)	0.001	2.08 (1.33–3.26)

For abbreviations see Table 1.

OR = odds ratio, CI = confidence interval.

### Prognostic value of change in hsTnT

A total of 35 patients (8.7%) died and 91 patients (22%) required rehospitalization for worsening HF during the follow-up period (280±240 days). Among the 35 deceased patients, 23 (66%) were HF-related or cardiovascular death.

**Fig 2:**
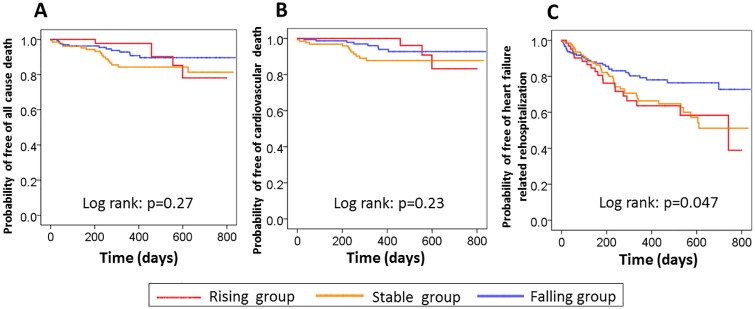
Comparison of Kaplan—Meier estimates for probability of (A) free of all cause death, (B) free of cardiovascular death, and (C) free of heart failure-related rehospitalization among the falling, stable, and rising groups. All cause death and cardiovascular death were not different among the falling, stable, and rising groups (Fig 2A, B). HF-related rehospitalization was significantly lower in falling group (p = 0.047; log-rank test; Fig 2C).

Kaplan-Meier analysis was applied for comparison of data of the falling, stable, and rising hsTnT groups, and those of the normalized and persistently abnormal groups. All-cause death and cardiovascular death were not different among the three groups (Fig 2A and 2B) and between the normalized and persistently abnormal group. HF-related rehospitalization was significantly lower in the falling group than the stable and rising groups (p = 0.047; log-rank test; Fig 2C) and lower in the normalized than the persistently abnormal group (p = 0.002; log-rank test). Univariate Cox hazard analysis showed that stable and rising hsTnT at discharge were associated with hazard ratio (HR) of 1.65 (95% CI: 1.03 to 2.63; p = 0.04) and HR of 1.34 (95% CI: 1.01 to 1.77; p = 0.04) for HF-related rehospitalization, compared with falling hsTnT at discharge. When included in the multivariate analysis model containing age, sex, atrial fibrillation, hemoglobin, serum hsTnT, BNP, serum sodium, creatinine levels on admission, and rising or stable hsTnT at discharge, rising or stable hsTnT at discharge as a significant predictor of HF-related rehospitalization (HR: 1.69; 95% CI: 1.06 to 2.70: p = 0.03; Table 4). When BNP level at discharge was included in instead of BNP level on admission, multivariate Cox hazard regression analysis identified that not rising or stable hsTnT at discharge but BNP level at discharge was a significant predictor of HF-related rehospitalization.

**Table 4:** Results of univariate and multivariate Cox proportional hazards regression analyses for heart-failure related rehospitalization.

Variables	Univariate Analysis		Multivariate Analysis	
	p value	HR (95% CI)	p value	HR (95% CI)
Age (per year)	0.004	1.03 (1.01–1.05)	0.04	1.03 (1.00–1.05)
Male	0.54	1.14 (0.74–1.75)	0.80	0.94 (0.60–1.48)
Hypertension	0.84	1.05 (0.67–1.64)		
Diabetes mellitus	0.25	1.28 (0.84–1.94)		
Dyslipidemia	0.48	1.16 (0.77–1.77)		
Atrial fibrillation	<0.001	2.61 (1.66–4.09)	0.002	2.16 (1.33–3.50)
Ischemic etiology	0.81	0.94 (0.57–1.54)		
Use of inotropic agents	0.20	1.40 (0.83–2.34)		
Use of carpertide	0.93	1.02 (0.67–1.55)		
LVEF (%)	0.56	1.00 (0.98–1.01)		
Duration of hospitalization (days)	0.65	1.00 (0.99–1.01)		
Hemoglobin (g/dl)	0.005	0.87 (0.78–0.96)	0.51	0.96 (0.85–1.08)
Serum hsTnT (ng/ml)	0.32	2.11 (0.49–9.09)	0.25	2.74 (0.50–14.97)
Plasma BNP (pg/ml)	0.10	1.00 (1.00–1.00)	0.02	1.00(1.00–1.00)
Serum sodium (mEq/L)	0.001	0.93 (0.89–0.97)	0.03	0.95 (0.91–1.00)
Estimated GFR (ml/min/1.73 m^2^)	<0.001	0.98 (0.97–0.99)		
Serum creatinine (mg/ml)	0.009	1.30 (1.07–1.59)	0.22	1.18 (0.91–1.52)
Total-bilirubin (mg/ml)	0.21	1.18 (0.91–1.52)		
hs-CRP (mg/ml)	0.27	1.03 (0.98–1.10)		
Rising or stable hsTnT (yes)	0.02	1.70 (1.10–2.62)	0.03	1.69 (1.06–2.70)
Increase in BNP (yes)	0.59	1.21 (0.61–2.40)		
Increase in creatinine (yes)	0.54	0.88 (0.58–1.33)		

HR = hazard ratio

For abbreviations see Tables 1 and 3.

## Discussion

The major findings of the present study were: *1)* Rising and stable hsTnT levels at discharge was noted in 36% and 17% of ADHF patients with abnormal hsTnT levels on admission and not hsTnT level on admission but the rising or stable hsTnT levels at discharge was a significant predictor of HF-related future hospitalization; *2)* Both the net and percent change in hsTnT correlated with changes in BNP level; *3)* The rising and stable hsTnT levels at discharge was associated with inadequate decrease of BNP levels and previous HF hospitalization. These findings indicate that insufficient improvement of cardiac wall stress seem to result in sustained myocardial damage and worse clinical outcome in patients with ADHF.

Several studies have measured the serial changes in cardiac troponin levels using conventional or high sensitivity assays during ADHF management [8–10,14]. They concluded that increased cardiac troponin level was associated with poor clinical outcome, similar to our results. In the RELAXin in Acute Heart Failure (RELAX-AHF) trial, Felker et al. [9] measured hsTnT levels at baseline and days 2, 5, and 14 in 1074 patients with ADHF. The median baseline hsTnT level was 0.033 ng/ml, which was comparable with our study. They concluded that baseline, peak, and peak change in hsTnT (largest change from baseline and peak hsTnT level) were associated with 180-day cardiovascular mortality. Our results are somewhat different from those of the above study. First, the above study evaluated hsTnT levels at scheduled time points (baseline and days 2, 5, and 14) and the change in hsTnT was defined as the difference between peak hsTnT at any point and baseline levels, which reflected the progression of myocardial damage during ADHF management. While the above study evaluated the precise time course of myocardial damage, it did not evaluate improvement of myocardial damage at discharge. Second, the above study analyzed cardiovascular death at 180 days and not rehospitalization at 180 days. Our study analyzed HF-related rehospitalization during longer follow-up period (280±240 days). Finally, baseline hsTnT levels were associated with worse outcome in REXAX-AHF trial. This result was in contrast to our results and results from Acute Study of Clinical Effectiveness of Nesiritide in Decompensated Heart Failure (ASCEND-HF) study, which demonstrated that baseline high-sensitive cardiac troponin I (hsTnI) levels were not associated with post-discharge outcomes at 30 days and 180 days [15]. Baseline hsTnT levels may be seen as urgency biomarker which reflects additional myocardial damage in patients with ADHF. Decrease of hsTnT levels (> -15%) predicted favorable prognosis compared to stable or rising hsTnT levels in our study. It may therefore be important to make a therapeutic intervention to achieve lower hsTnT levels at discharge in patients with ADHF, even if baseline hsTnT levels are high.

Xue et al. [10] also conducted serial measurements of hsTnI levels (on admission, up to four consecutive days during hospitalization, and at discharge) in 106 ADHF patients. Among them, 65 (61%) showed increases in hsTnI (defined as peak hsTnI at any point after admission) during hospitalization. They concluded that early increase in hsTnI during hospitalization was associated with increased 90-day mortality and readmission. Compared to their study, we focused on improvement of myocardial damage at discharge. Moreover, our study included larger number of patients with longer follow-up period. Our results add further support to the prognostic significance of relief of myocardial damage in patients with ADHF.

The exact mechanism responsible for cardiac troponin release from the myocardium in patients with HF remains speculative. Various reasons have been proposed for the persistent hypertroponinemia, such as myocardial ischemia with or without coronary artery disease, increased wall stress, myocyte damage from inflammatory cytokines and/or oxidative stress, neurohormonal activation and coronary microvascular dysfunction [1,6]. These potential mechanisms are activated in ADHF compared to compensated state and enhance cardiac troponin release through myocardial necrosis, apoptosis, and troponin degradation [16,17]. Since BNP is released from the heart in response to volume expansion and pressure overload associated with wall stress [18], it is assumed that decreases in BNP levels in patient with ADHF are associated with relief of myocardial damage. In our study, changes in hsTnT levels were correlated with changes in BNP levels. However, such correlation was weak (ρ = 0.221) and the hsTnT level at discharge was increased in about one-third of ADHF patients, while plasma BNP level was decreased at discharge in the majority of study patients. These results suggest that increase in wall stress is probably only one of several mechanisms of cardiac troponin release in ADHF.

Rehospitalization due to ADHF is a social and economic problem in developed countries [19]. Repeat decompensation leads to further impairment of myocardial function, and worsening of myocardial dysfunction exacerbates HF-related symptoms and activates various factors associated with myocardial damage [7]. In this vicious cycle of worsening HF, myocardial damage might become irreversible and progressive, leading to poor clinical outcome. Therapeutic strategies and optimal medical therapy for relief of myocardial damage during ADHF management have not been fully identified. Measurement of change in hsTnT could be useful to evaluate improvement of myocardial damage due to therapeutic intervention to the several hypertroponinemia-related factors, such as myocardial ischemia or neurohormonal activation. Further research is warranted to elucidate therapeutic strategies that can reduce both cardiac troponin (myocardial damage) and BNP (hemodynamics) levels during ADHF management to improve clinical outcome.

### Study limitations

Our study has several limitations that need to be taken into account. First, the study was conducted using data from a prospective registry at a single institution. Further multicenter studies of larger groups are needed to confirm the present results. Second, our study population consisted of fewer ischemic etiology (21%) compared to the patients in acute decompensated heart failure syndromes (ATTEND) registry (31%), which is the largest nationwide, multicenter, prospective cohort study in Japan [20], and several large ADHF registries and trials in Europe and United States (50–60%) [3,8,10,21,22]. One of the reasons was the exclusion in our study of patients who underwent revascularization procedure or surgical intervention during hospitalization (n = 22). Because myocardial ischemia is one of the major factors that affect cardiac troponin release, the presence of coronary artery disease or unrecognized acute coronary syndrome could influence the change in cardiac troponin. Further studies are warranted to determine the change in cardiac troponin in ADHF patients with or without overt coronary artery disease. Finally, serum hsTnT levels are affected by renal dysfunction [23]. Therefore, it is considered that impaired renal function during ADHF management could indirectly cause a rise in hsTnT.

## Conclusions

Persistent increase in hsTnT at discharge was observed in about half of ADHF patients and was a significant and independent predictor of future HF-related readmission to the hospital. Rising and stable hsTnT levels at discharge correlated with inadequate decrease of BNP levels at discharge and previous HF hospitalization. These results suggest that inadequate improvement of cardiac wall stress can be associated with persistent myocardial damage and repeat HF hopistalization in patients with ADHF. Thus, therapeutic or preventive interventions aimed at reducing myocardial damage might help to break the vicious cycle of worsening HF syndrome.

## Supporting information

S1 File Dataset of this study. (XLSX)
